# Innovative Approach of Non-Thermal Plasma Application for Improving the Growth Rate in Chickens

**DOI:** 10.3390/ijms19082301

**Published:** 2018-08-06

**Authors:** Jiao Jiao Zhang, Xian Zhong Wang, Taeho Kwon, Do Luong Huynh, Nisansala Chandimali, Nameun Kim, Tae Yoon Kang, Mrinmoy Ghosh, Meeta Gera, Sang Baek Lee, Sung Jin Lee, Wang Shik Lee, Seong Bong Kim, Young Sun Mok, Dong Kee Jeong

**Affiliations:** 1 Laboratory of Animal Genetic Engineering and Stem Cell Biology, Department of Advanced Convergence Technology and Science, Jeju National University, Jeju 63243, Korea; zhangjjff@126.com (J.J.Z.); denishhuynh@gmail.com (D.L.H.); nimminisha28@gmail.com (N.C.); nameun82@gmail.com (N.K.); tyfy3@nate.com (T.Y.K.); mringhs2010@gmail.com (M.G.); mee21988@gmail.com (M.G.); 2 Chongqing Key Laboratory of Forage and Herbivore, College of Animal Science and Technology, Southwest University, Chongqing 400715, China; xianzhong_wang@aliyun.com; 3 Laboratory of Animal Genetic Engineering and Stem Cell Biology, Subtropical/Tropical Organism Gene Bank, Jeju National University, Jeju 63243, Korea; taehokwonk@gmail.com; 4 Department of Chemical and Biological Engineering, Jeju National University, Jeju 63243, Korea; sblee@jejunu.ac.kr; 5 Department of Animal Biotechnology, College of Animal Bioscience and Technology, Kangwon National University, Chunchon 24341, Korea; Sjlee@kangwon.ac.kr; 6 Department of Animal Biotechnology, College of Applied Life Sciences, Jeju National University, Jeju 63243, Korea; leewshik@naver.com; 7 Plasma Technology Research Center, National Fusion Research Institute, Gunsan-si, Jeollabuk-Do 54004, Korea; sbkim@nfri.re.kr

**Keywords:** non-thermal plasma, growth metabolism, demethylation, ATP, GH-IGF1, thyroid hormones

## Abstract

As an innovative technology in biological applications—non-thermal plasma technique—has recently been applied to living cells and tissues. However, it is unclear whether non-thermal plasma treatment can directly regulate the growth and development of livestock. In this study, we exposed four-day-incubated fertilized eggs to plasma at 11.7 kV for 2 min, which was found to be the optimal condition in respect of highest growth rate in chickens. Interestingly, plasma-treated male chickens conspicuously grew faster than females. Plasma treatment regulated the reactive oxygen species homeostasis by controlling the mitochondrial respiratory complex activity and up-regulating the antioxidant defense system. At the same time, growth metabolism was improved due to the increase of growth hormone and insulin-like growth factor 1 and their receptors expression, and the rise of thyroid hormones and adenosine triphosphate levels through the regulation of demethylation levels of growth and hormone biosynthesis-related genes in the skeletal muscles and thyroid glands. To our knowledge, this study was the first to evaluate the effects of a non-thermal plasma treatment on the growth rate of chickens. This safe strategy might be beneficial to the livestock industry.

## 1. Introduction

Non-thermal plasma systems have the potential for a wide-range of biological applications in living cells and tissues [1] because of no substantial gas heating. Our laboratory has established a non-thermal dielectric barrier discharge (DBD) plasma system generated in argon at atmospheric pressure for creating electrically safe plasma [2,3,4,5,6]. A previous study suggested that an appropriate non-thermal DBD plasma treatment improved the seed germination and sprout growth in soybeans [5]. However, it is unclear whether non-thermal plasma treatment can directly regulate the growth and development of livestock. In this study, the non-thermal DBD plasma technique was implemented in fertilized eggs before hatching for the evaluation of the optimal plasma treatment conditions. In vitro experiments using fibroblasts [7], endothelial cells [8], epithelial cells [1], and myoblast cells [9] demonstrate a plasma dose-dependent effect. These previous studies suggested that plasma intensity and exposure time could result in differentiation of growth-promoting effects in chickens.

Numerous small pores in the palisades of chicken eggshell permit the potential diffusion of active charged and neutral particles in plasma, which generates reactive oxygen species (ROS) when exposed on the surface of cells or tissues [10]. Diffusion of plasma-produced ROS or accumulation of plasma-stimulated intracellular ROS [11,12] regulate cell proliferation and differentiation [8,13] and mitochondrial biogenesis [14]. Non-thermal plasma-produced appropriate extracellular ROS increases skeletal cell differentiation and limb development of mouse through the activation and amplification of intracellular ROS-sensitive signaling pathways [15]. However, excessive accumulation of intracellular ROS induces cell apoptosis [6,16,17]. In this study, we try to investigate whether plasma-produced ROS or stimulation of intracellular ROS mediate the chicken growth.

A genome-wide DNA methylation map in chickens displayed analogous hallmarks of classic vertebrate patterns [18]. DNA methylation is negatively correlated with gene transcription in birds [18]. DNA hypomethylation generally correlates with the activation of gene expression and acceleration of metabolic activity [19]. However, hypermethylation suppresses gene expression by disturbing the binding of specific transcription factors [20]. DNA methylation levels of differentially methylated cytosine guanine (CG) sites are correlated with the relative maternal stress levels during pregnancy [21]. Thus, we hypothesize that the non-thermal DBD plasma treatment on fertilized chicken eggs may change the DNA methylation levels during the developmental process. Furthermore, sex differences at the DNA methylation level have previously been studied in skeletal muscles [22]. Chromosome-wide and gene-specific sex differences in DNA methylation are associated with differential gene expression and metabolism [23]. Females tend to have greater DNA methylation levels on both the allosomes and autosomes than males [24]. As a result, we predict that plasma may induce changes in methylation levels that differ between female and male chickens.

The aim of the present study was therefore to determine whether non-thermal DBD plasma treatment can affect the chicken growth. We also investigate whether the plasma effect differs by sex and how plasma regulates energy metabolism during the developmental process.

## 2. Results

### 2.1. Optimization of Plasma Treatment Condition on the Chicken Growth

**Figure 1:**
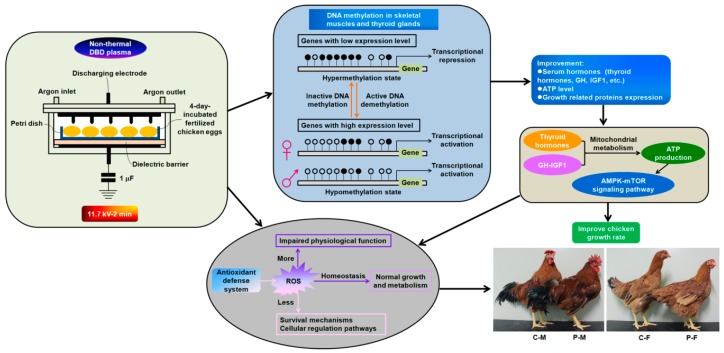
Description of the mechanism of how non-thermal dielectric barrier discharge (DBD) plasma affect chicken growth. The optimal plasma treatment condition is 11.7 kV for 2 min. Representative chicken growth status on day 90 is shown. C-M represents male chickens in the control group; P-M represents male chickens in the plasma treatment group. C-F represents female chickens in the control group; P-F represents female chickens in the plasma treatment group. The antioxidant defense system-mediated reactive oxygen species (ROS) homeostasis regulates the growth performance. DNA methylation in skeletal muscles and thyroid glands is involved in the regulation of chicken growth rate. ● represent methylated cytosine, and ○ represent unmethylated cytosine.

Chickens in the group exposed to 22.0 kV for 1 min showed the highest average daily gain (ADG) and longest average tibia length among all plasma-treated groups in the first month (Table 1). The ADG and tibia length of chickens exposed to 11.7 kV for 2 min exhibited the maximum values among all plasma-treated groups at the age of 90 days, with increases of 0.15- and 0.11-fold (*p <* 0.001) in females and 0.23- and 0.12-fold (*p <* 0.001) in males, respectively, when compared with the females and males of the control group (Table 1). The photographs of 90-day-old chickens in the plasma group exposed to 11.7 kV for 2 min and the control group were shown in Figure 1. Interestingly, plasma-treated male chickens showed conspicuously faster growth rate than females (Table 1). Higher potential plasma (27.6 kV) produced inhibitory effects on chicken growth parameters (Table 1). Thus, non-thermal DBD plasma exposure at 11.7 kV for 2 min was used as the optimal condition for further experiments.

### 2.2. Chicken Serum Hormone Levels

**Figure 2:**
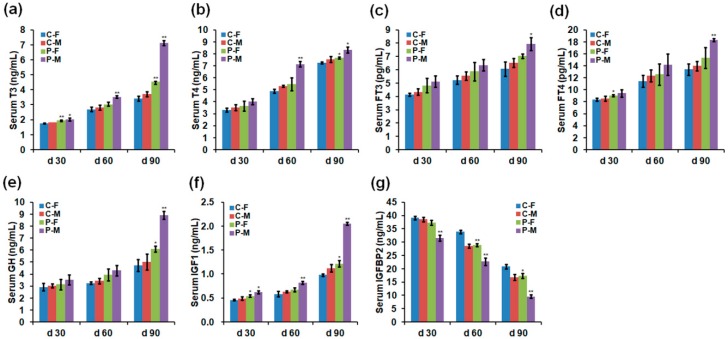
Chicken serum hormone levels on days 30, 60, and 90. (**a**) triiodothyronine (T3), (**b**) thyroxine (T4), (**c**) free triiodothyronine (FT3), (**d**) free thyroxine (FT4), (**e**) growth hormone (GH), (**f**) insulin-like growth factor 1 (IGF1), and (**g**) insulin-like growth factor binding protein 2 (IGFBP2). Data are presented as mean ± SD (*n* = 10) of three replicates; n represents an individual chicken. *, *p <* 0.05 versus control; **, *p <* 0.01 versus control in females and males, respectively, on days 30, 60, and 90, according to the one-way ANOVA with an LSD test.

Compared to 30-day-old females and males in the control group, plasma exposure at 11.7 kV for 2 min significantly increased the serum concentrations of triiodothyronine (T3) in both females and males (Figure 2a), free thyroxine (FT4) in females (Figure 2d), and insulin-like growth factor 1 (IGF1) in both females and males (Figure 2f), but decreased insulin-like growth factor binding protein 2 (IGFBP2) in males (Figure 2g). Compared to 60-day-old females and males in the control group, plasma exposure significantly increased the serum concentrations of T3, thyroxine (T4), and IGF1 in males (Figure 2a,b,f), but decreased IGFBP2 in both females and males (Figure 2g). Compared to 90-day-old females and males in the control group, plasma exposure significantly increased the serum concentrations of T3 and T4 in both females and males (Figure 2a,b), free triiodothyronine (FT3) and FT4 in males (Figure 2c,d), and growth hormone (GH) and IGF1 in both females and males (Figure 2e,f), but decreased IGFBP2 in both females and males (Figure 2g). Specifically, male chickens showed greater changes in those serum hormone levels than female chickens after plasma treatment. The detailed parameters on day 90 showed that plasma treatment increased 0.32- (*p =* 0.001) and 0.91-fold (*p <* 0.001) of T3 in females and males (Figure 2a), 0.06- (*p =* 0.011) and 0.10-fold (*p =* 0.033) of T4 in females and males (Figure 2b), 0.16- (*p =* 0.071) and 0.21-fold (*p =* 0.028) of FT3 in females and males (Figure 2c), 0.15- (*p =* 0.235) and 0.31-fold (*p =* 0.002) of FT4 in females and males (Figure 2d), 0.29- (*p =* 0.023) and 0.78-fold (*p =* 0.002) of GH in females and males (Figure 2e), and 0.24- (*p =* 0.011) and 0.83-fold (*p <* 0.001) of IGF1 in females and males (Figure 2f), respectively, compared to 90-day-old females and males in the control group. However, the serum IGFBP2 level showed a 0.17- (*p =* 0.016) and 0.43-fold (*p =* 0.001) decrease in 90-day-old females and males after plasma exposure (Figure 2g).

### 2.3. Adenosine Triphosphate (ATP), ROS, and Antioxidant Enzyme Levels

**Figure 3:**
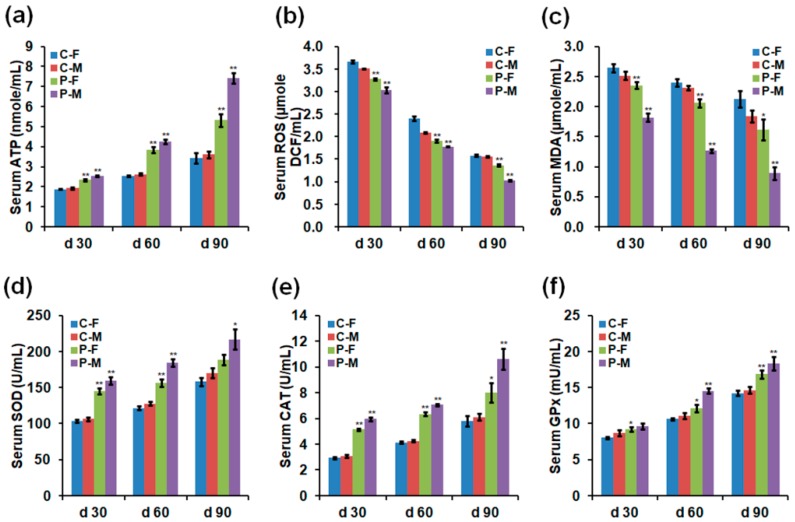
Concentrations of (**a**) adenosine triphosphate (ATP), (**b**) ROS, (**c**) malondialdehyde (MDA), (**d**) superoxide dismutase (SOD), (**e**) catalase (CAT), and (**f**) glutathione peroxidase (GPx) in the serum of chickens on days 30, 60, and 90. Data are presented as mean ± SD (*n* = 10) of three replicates; n represents an individual chicken. *, *p <* 0.05 versus control; **, *p <* 0.01 versus control in females and males, respectively, on days 30, 60, and 90, according to the one-way ANOVA with an LSD test.

**Figure 4:**
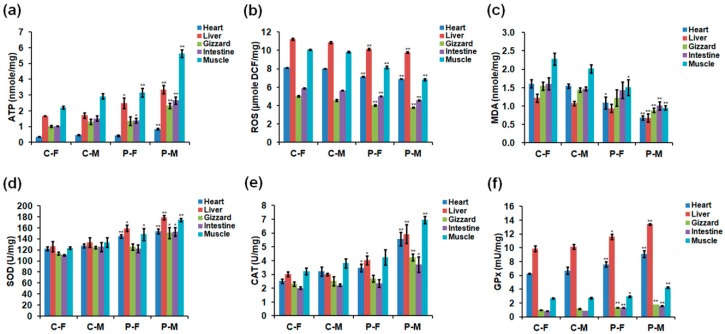
Concentrations of (**a**) ATP, (**b**) ROS, (**c**) MDA, (**d**) SOD, (**e**) CAT, and (**f**) GPx in different organs of 90-day-old chickens. Data are presented as mean ± SD (*n* = 10) of three replicates; n represents an individual chicken. *, *p <* 0.05 versus control; **, *p <* 0.01 versus control in females and males, respectively, according to the one-way ANOVA with an LSD test.

Optimal plasma exposure significantly increased the concentrations of ATP, superoxide dismutase (SOD), catalase (CAT), and glutathione peroxidase (GPx) but reduced ROS and malondialdehyde (MDA) levels in chicken sera on days 30, 60, and 90 (Figure 3) and in different organs on day 90 (Figure 4). The detailed parameters revealed that males showed that values of ATP, ROS, and antioxidant enzymes varied in a greater extent than females after plasma treatment (Figure 3 and Figure 4). In terms of ATP level on day 90, plasma-treated females and males showed increases of 0.55- (*p =* 0.003) and 1.06-fold (*p <* 0.001) in the serum (Figure 3a), 0.19- (*p =* 0.101) and 0.83-fold (*p =* 0.001) in the heart, 0.48- (*p =* 0.027) and 0.96-fold (*p =* 0.002) in the liver, 0.32- (*p =* 0.172) and 0.77-fold (*p =* 0.004) in the gizzard, 0.34- (*p =* 0.027) and 0.75-fold (*p =* 0.004) in the intestine, and 0.43- (*p =* 0.009) and 0.92-fold (*p <* 0.001) in the skeletal muscle (Figure 4a), respectively, compared to 90-day-old females and males in the control group. However, ROS level of females and males on day 90 were negatively affected by plasma treatment, showing decreases of 0.13- (*p =* 0.001) and 0.34-fold (*p <* 0.001) in the serum (Figure 3b), 0.12- (*p <* 0.001) and 0.14-fold (*p <* 0.001) in the heart, 0.09- (*p <* 0.001) and 0.10-fold (*p <* 0.001) in the liver, 0.15- (*p =* 0.004) and 0.17-fold (*p =* 0.001) in the gizzard, 0.14- (*p <* 0.001) and 0.19-fold (*p <* 0.001) in the intestine, and 0.19- (*p <* 0.001) and 0.31-fold (*p <* 0.001) in the skeletal muscle (Figure 4b), respectively, compared to those in the controls. Moreover, MDA level of females and males on day 90 were also negatively affected by plasma treatment, showing decreases of 0.24- (*p =* 0.031) and 0.52-fold (*p =* 0.001) in the serum (Figure 3c), 0.32- (*p =* 0.021) and 0.56-fold (*p <* 0.001) in the heart, 0.23- (*p =* 0.065) and 0.37-fold (*p =* 0.010) in the liver, 0.20- (*p =* 0.143) and 0.38-fold (*p =* 0.001) in the gizzard, 0.11- (*p =* 0.420) and 0.31-fold (*p =* 0.006) in the intestine, and 0.34- (*p =* 0.015) and 0.53-fold (*p <* 0.001) in the skeletal muscle (Figure 4c), respectively. In terms of SOD activity on day 90, plasma-treated females and males showed increases of 0.19- (*p =* 0.100) and 0.27-fold (*p =* 0.013) in the serum (Figure 3d), 0.18- (*p =* 0.002) and 0.20-fold (*p =* 0.002) in the heart, 0.26- (*p =* 0.012) and 0.34-fold (*p =* 0.003) in the liver, 0.11- (*p =* 0.052) and 0.21-fold (*p =* 0.024) in the gizzard, 0.11- (*p =* 0.077) and 0.22-fold (*p =* 0.028) in the intestine, and 0.20- (*p =* 0.035) and 0.31-fold (*p =* 0.004) in the skeletal muscle (Figure 4d), respectively, compared to those in the controls. In terms of CAT activity on day 90, plasma-treated females and males showed increases of 0.38- (*p =* 0.021) and 0.74-fold (*p =* 0.002) in the serum (Figure 3e), 0.38- (*p =* 0.015) and 0.73-fold (*p =* 0.005) in the heart, 0.33- (*p =* 0.018) and 0.97-fold (*p =* 0.004) in the liver, 0.17- (*p =* 0.132) and 0.68-fold (*p =* 0.004) in the gizzard, 0.17- (*p =* 0.158) and 0.68-fold (*p =* 0.019) in the intestine, and 0.32- (*p =* 0.073) and 0.83-fold (*p <* 0.001) in the skeletal muscle (Figure 4e), respectively, compared to those in the controls. In addition, GPx activity of females and males on day 90 were increased by plasma treatment, with increases of 0.18- (*p =* 0.006) and 0.25-fold (*p =* 0.07) in the serum (Figure 3f), 0.21- (*p =* 0.008) and 0.36-fold (*p =* 0.009) in the heart, 0.17- (*p =* 0.013) and 0.32-fold (*p <* 0.001) in the liver, 0.36- (*p <* 0.001) and 0.57-fold (*p <* 0.001) in the gizzard, 0.61- (*p <* 0.001) and 0.81-fold (*p <* 0.001) in the intestine, and 0.11- (*p =* 0.024) and 0.55-fold (*p <* 0.001) in the skeletal muscle (Figure 4f), respectively, compared to those in the controls.

### 2.4. Ultrastructure of Skeletal Muscles and Mitochondrial Respiratory Enzyme Levels in Male Chickens

**Figure 5:**
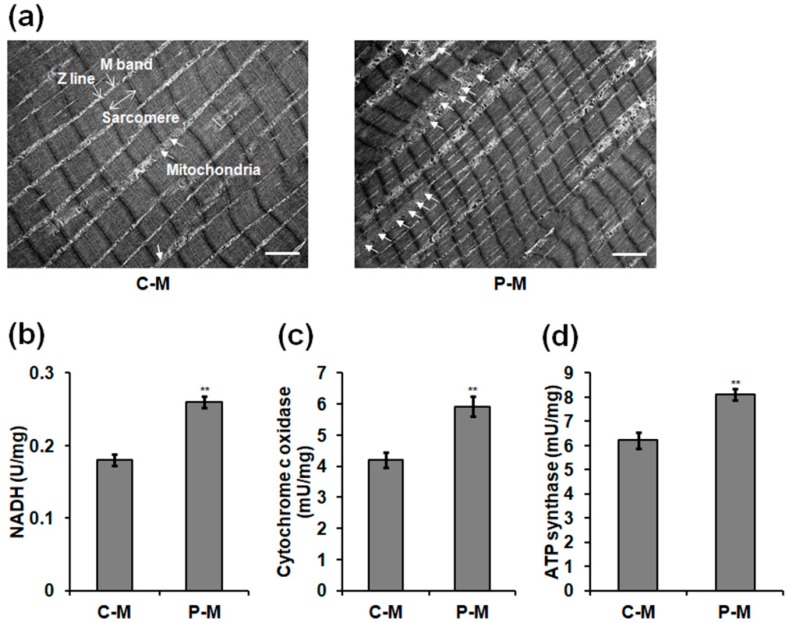
Ultrastructure of skeletal muscles and mitochondrial respiratory enzyme levels in 90-day-old male chickens. (**a**) Representative longitudinal section of skeletal muscles. Z line, M band, and sarcomere in the myofibril are clearly observed. Mitochondria located between myofibrils are photographed using a transmission electron microscopy. The arrow shows the mitochondrion. Scale bar: 2.0 μm. (**b**) Nicotinamide adenine dinucleotide hydrogen (NADH) levels in the mitochondria of skeletal muscles. Activities of mitochondrial respiratory enzyme (**c**) cytochrome c oxidase and (**d**) ATPase synthase in skeletal muscles. Data are presented as mean ± SD (*n* = 10) of three replicates; n represents an individual chicken. **, *p <* 0.01 versus control, according to the one-way ANOVA with an LSD test.

Considering greater growth parameters presented in the plasma-treated male chickens, we investigated the ultrastructure of skeletal muscles and mitochondrial respiratory complex activity in skeletal muscles of males. The results showed that the number of mitochondria present between the myofibrils in the longitudinal section of the skeletal muscles of plasma-treated male chickens aged 90 days was greater than in the control group (Figure 5a). The optimal plasma exposure increased the levels of nicotinamide adenine dinucleotide hydrogen (NADH) (Figure 5b), cytochrome c oxidase (Figure 5c), and ATP synthase (Figure 5d) by 0.44- (*p =* 0.001), 0.30- (*p =* 0.003), and 0.41-fold (*p =* 0.004) in the skeletal muscle mitochondria of 90-day-old male chickens.

### 2.5. Expressions of mRNA and Protein in the Skeletal Muscles

**Figure 6:**
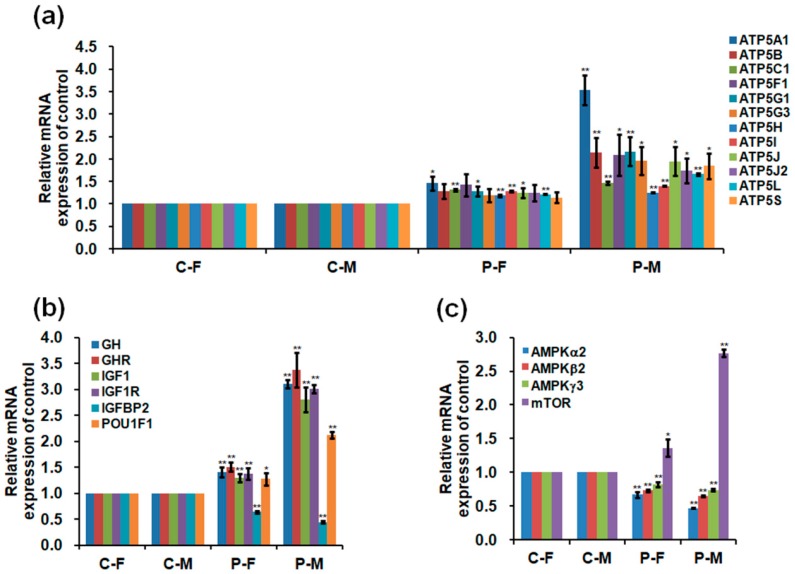

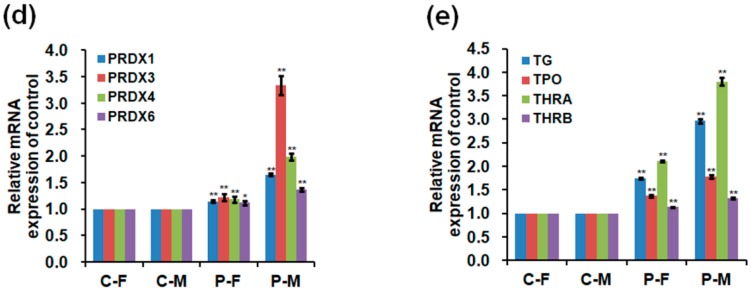
Relative mRNA levels of (**a**) *ATP5* synthase; (**b**) *GH*, growth hormone receptor (*GHR*), *IGF1*, insulin-like growth factor 1 receptor (*IGF1R*), *IGFBP2*, and POU class 1 homeobox 1 (*POU1F1*); (**c**) adenosine monophosphate-activated protein kinase (*AMPK*) *α2*, *AMPKβ2*, *AMPKγ3*, and mammalian target of rapamycin (*mTOR*), and (**d**) peroxiredoxin (*PRDX*) *1*, *PRDX3*, *PRDX4*, and *PRDX6* in the skeletal muscles of 90-day-old chickens; Relative mRNA levels of thyroglobulin (*TG*), thyroid peroxidase (*TPO*), thyroid hormone receptor alpha (*THRA*), and thyroid hormone receptor beta (*THRB*) (**e**) in the thyroid glands of 90-day-old chickens. Data are presented as mean ± SD (*n* = 10) of three replicates; n represents an individual chicken. *, *p <* 0.05 versus control; **, *p <* 0.01 versus control in females and males, respectively, according to the one-way ANOVA with an LSD test.

**Figure 7:**
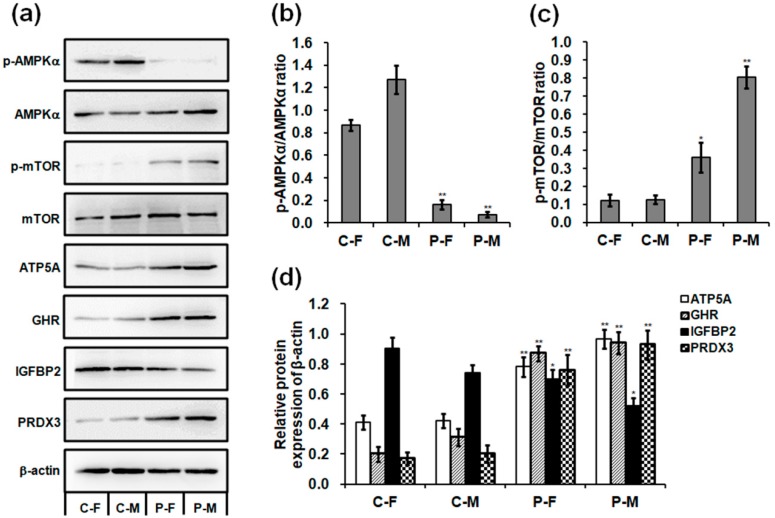
Effect of plasma on protein expression in 90-day-old chickens. (**a**) Western blot analysis of protein bands in the skeletal muscles. Uncropped immunoblot scans are presented in Figure S1. Relative protein levels of (**b**) p-AMPKα/AMPKα, (**c**) p-mTOR/mTOR, and (**d**) ATP5A, GHR, IGFBP2, and PRDX3. Data are presented as mean ± SD (*n* = 3) of three replicates; *n* represents an individual chicken. *, *p <* 0.05 versus control; **, *p <* 0.01 versus control in females and males, respectively, according to the one-way ANOVA with an LSD test.

The optimal plasma exposure significantly increased the mRNA expression of *ATP5* synthase, *GH*, growth hormone receptor (*GHR*), *IGF1*, insulin-like growth factor 1 receptor (*IGF1R*), POU class 1 homeobox 1 (*POU1F1*), mammalian target of rapamycin (*mTOR*), and peroxiredoxins (*PRDXs*) but decreased the mRNA expression of *IGFBP2* and adenosine monophosphate-activated protein kinase (*AMPK*) in the skeletal muscle of chickens on day 90 (Figure 6a–d). In addition, plasma treatment increased the mRNA expression of thyroglobulin (*TG*), thyroid peroxidase (*TPO*), and thyroid hormone receptors (*THRs*) in the thyroid glands of chickens on day 90 (Figure 6e). Plasma treatment activated mTOR phosphorylation and increased the protein expression of ATP5A, GHR, and PRDX3 in the skeletal muscles, with increases of 1.99- (*p =* 0.018) and 5.43-fold (*p <* 0.001) in mTOR phosphorylation (Figure 7a,c); 0.90- (*p =* 0.003) and 1.29-fold (*p =* 0.001) in ATP5A (Figure 7a,d); 3.34- (*p <* 0.001) and 2.03-fold (*p =* 0.001) in GHR (Figure 7a,d); and 3.42- (*p =* 0.002) and 3.62-fold (*p =* 0.001) in PRDX3 (Figure 7a,d) in the skeletal muscle of female and male chickens compared to those in the controls. However, plasma treatment decreased AMPKα phosphorylation by 0.81- (*p <* 0.001) and 0.94-fold (*p <* 0.001) and IGFBP 2 protein expression by 0.23- (*p =* 0.042) and 0.30-fold (*p =* 0.012) in the skeletal muscle of female and male chickens (Figure 7a,b,d). Uncropped immunoblots in the skeletal muscle of chickens are presented in Figure S1. Interestingly, males exhibited greater changes in mRNA levels of the examined enzymes than females after plasma treatment.

### 2.6. DNA Methylation Level

**Figure 8:**
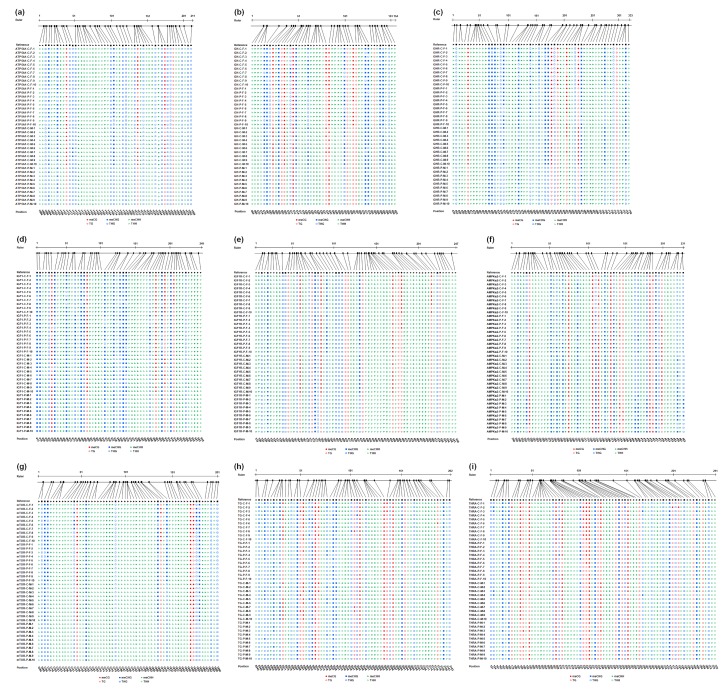
Cytosine methylation analysis in 90-day-old chickens. Bisulfite sequencing results of (**a**) *ATP5A1*, (**b**) *GH*, (**c**) *GHR*, (**d**) *IGF1*, (**e**) *IGF1R*, (**f**) *AMPKα2*, and (**g**) *mTOR* in the skeletal muscles, (**h**) *TG*, and (**i**) *THRA* in the thyroid glands. The length of sequenced region and exact location of cytosine are shown schematically. The order of individual sequences of ten clones is listed on the left. Reference sequence is shown in the first line. The sequence is distinguished by circles for cytosine guanine (CG), squares for CHG (where H correspond to adenosine, thymine, or cytosine), and triangles for CHH. Filled symbols represent methylated cytosine, and open symbols represent unmethylated cytosine.

**Figure 9:**
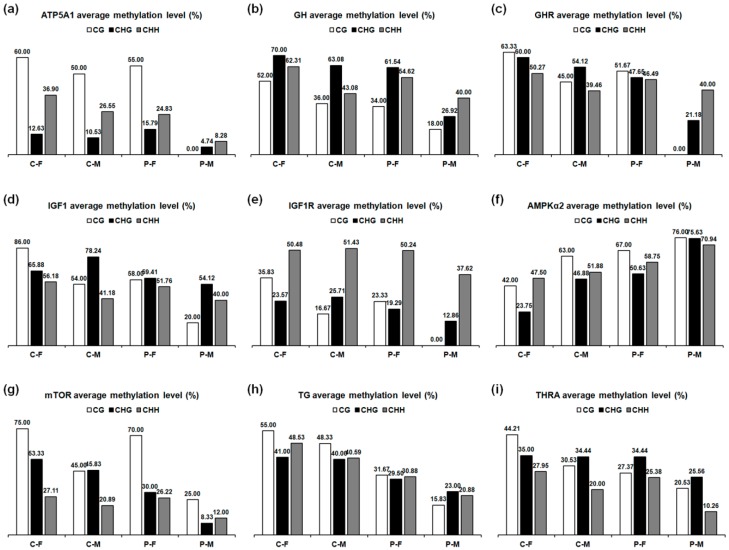
Average methylation levels for CG, CHG, and CHH sites in 90-day-old chickens. Average methylation levels in the sequenced regions of (**a**) *ATP5A1*, (**b**) *GH*, (**c**) *GHR*, (**d**) *IGF1*, (**e**) *IGF1R*, (**f**) *AMPKα2*, and (**g**) *mTOR* in the skeletal muscles, (**h**) *TG*, and (**i**) *THRA* in the thyroid glands.

Bisulfite sequencing of *ATP5A1*, *GH*, *GHR*, *IGF1*, *IGF1R*, *AMPKα2*, *mTOR*, *TG*, *THRA* was performed to determine the exact location, type, and extent of methylation. The sequence analysis results of these genes using CyMATE software are reported in Figure 8. Methylation levels in the sequenced regions of genes in the plasma-treated group decreased in the skeletal muscle of females and males on day 90 by, in corresponding order, 0.06- and 0.16-fold in *ATP5A1*, 0.10- and 0.15-fold in *GH*, 0.07- and 0.14-fold in *GHR*, 0.07- and 0.11-fold in *IGF1*, 0.03- and 0.14-fold in *IGF1R*, and 0.06- and 0.15-fold in *mTOR*. Methylation levels decreased by 0.17- and 0.21-fold in *TG* and 0.06- and 0.10-fold in *THRA* in the thyroid gland of females and males compared to those in the controls (Table 2). However, plasma treatment increased methylation levels in the sequenced region of *AMPKα2* by 0.18- and 0.21-fold in the skeletal muscle of females and males (Table 2). The decrease in average methylation levels for CG, CHG (where H correspond to adenosine, thymine, or cytosine), and CHH sites in the sequenced regions of *ATP5A1*, *GH*, *GHR*, *IGF1*, *IGF1R*, *mTOR*, *TG*, and *THRA* and the increase in average methylation levels for each type of *AMPKα2* in males were greater than those in females after plasma treatment (Figure 9).

## 3. Discussion

As an innovative technology in biological applications, non-thermal DBD plasma has recently been developed for application in the treatment of wounds, cancers, dental decays, and dermatological indications, and enhancement in the cell transfection efficiency, cell proliferation, and tissue regeneration [25]. In the present study, we exposed four-day-incubated fertilized eggs to plasma at 11.7 kV for 2 min, which was used as the optimal treatment condition in respect of highest growth rate in chickens. As shown, higher potential plasma (27.6 kV) produced inhibitory effects on chicken growth parameters. Our previous study confirmed that high doses of non-thermal plasma exposure resulted in chicken embryonic death but appropriate treatment promoted chicken embryonic development during the early stage of incubation [4]. The findings that low-intensity plasma enhanced cell proliferation [1,8] and limb growth [15] but high doses resulted in anti-proliferation [7,9] are supported by the present study leading to the conclusion that an appropriate plasma treatment should be optimized for promoting the chicken growth.

Thyroid hormones play an important role in the skeletal cell differentiation and growth [26]. DNA hypomethylation generally activates gene transcription [19] but hypermethylation suppresses gene expression [20], which mediate body mass index in the development process [27]. We found optimal plasma treatment up-regulated the mRNA expression of *TG*, *TPO*, and *THRs* with hypomethylation levels of *TG* and *THRA* in the thyroid glands, resulting in significant increases of serum T3, T4, FT3, and FT4, which contribute to stimulate the energy production and protein synthesis. In this study, plasma treatment enhanced serum GH and IGF1 levels, which contribute to improve the energy metabolism and protein synthesis for muscle and bone growth in poultry [28,29], but reduced serum IGFBP2 and its protein level in the skeletal muscles, which inhibit cell growth by preventing IGF1 binding to its receptor [29], through increasing the *GH* and *IGF1* mRNA expression and decreasing the *IGFBP2* mRNA expression. In addition, plasma treatment up-regulated the *GHR* mRNA level, *IGF1R* mRNA and its demethylation levels, and *POU1F1* mRNA level that stimulates GH synthesis [30] in the skeletal muscles. These findings inferred that non-thermal plasma treatment stimulated a signaling cascade in the GH-IGF1 and thyroid hormones by regulating the demethylation levels of *TG*, *THRA*, *GH*, *GHR*, *IGF1*, and *IGF1R*, for the improvement of chicken growth.

GH-IGF1 signaling and thyroid hormones stimulate the muscle mitochondrial metabolism by increasing the oxidative enzyme activity and ATP production [31,32,33]. In this study, plasma treatment increased the number of mitochondria that contribute to increase the ATP production and energy metabolism [34] and respiratory complex activity that is a major contributor to the whole-body respiration and energy expenditure [35] in the skeletal muscles of chickens. In addition, plasma treatment improved the ATP production through increasing the *ATP* synthase subunit mRNA expression with a hypomethylation level of *ATP5A1* and ATP5A protein. Furthermore, high concentrations of ATP inactivate AMPK, which contributes to activate mTOR pathway [36], resulting in the improvement of energy metabolism [37]. In this study, the increased daily gain of chickens exposed to plasma revealed a strong signaling correlation between the activated state of mTOR phosphorylation and opposed p-AMPK. Therefore, plasma treatment improves the ATP production and energy metabolism, which are regulated by the signaling cascade in GH-IGF1 and thyroid hormones, the increase in *ATP5A1* demethylation, and the AMPK-mTOR signaling pathway.

Mitochondrial respiratory chain produces cellular ROS [38] and influences the physiological levels of ROS in the plasma-induced oxidative stress [39]. The balance between ROS production and scavenging activity which can be regulated by the catabolism in antioxidant enzymes [12,40,41] influences the cell proliferation and differentiation [8,13]. Our study found optimal plasma treatment controlled intracellular ROS relatively low and significantly reduced MDA activity, which were mediated by up-regulating antioxidant enzyme levels of SOD, CAT, and GPx and *PRDX* mRNA expression and PRDX3 protein levels. Thyroid hormones can increase antioxidant defense system in different tissues [42] and GH-IGF1 signaling also can increase antioxidant enzyme activity [43,44]. Our findings revealed that the appropriate non-thermal plasma treatment regulates ROS balance for improving the chicken growth through influencing the mitochondrial respiratory complex activity and up-regulating the antioxidant enzyme activity which was mediated by thyroid hormones and GH-IGF.

This study found plasma-treated male chickens significantly grew faster than plasma-treated females. Sex differences at the DNA methylation which are associated with differential gene expression and metabolism [23] have previously been studied in skeletal muscles [22]. Females tend to have greater DNA methylation levels than males on both the allosomes and the autosomes [24]. In this study, demethylation level increase of the growth and hormone biosynthesis-related genes in the male chickens after plasma exposure were greater than those in females, and were correlated with greater mRNA expression levels of those genes in males because the demethylation has been associated with the transcriptional activation of selected imprinted genes [45], resulting in greater serum hormone levels, ATP levels, and protein expressions in skeletal muscles of male chickens, thereby inducing faster growth of males than of females after plasma treatment.

## 4. Materials and Methods

### 4.1. Plasma Treatment and Chicken Growth

This study was carried out in strict accordance with the Animal Care and Use Committee of Jeju National University (approval number: 2016-0022, 22 January 2016) and the Institutional Committee for Ethics in Animal Experiments of Jeju National University. Artificial insemination was performed twice per week with semen collected from the cockerels (*Korean native chicken*, commercially used for broiler) which were raised at a chicken farm in Jeju National University, Jeju, Republic of Korea. Fertilized eggs (70 eggs for each group) obtained from hens (*Hyline brown chicken*, layer) were incubated at 37.5 °C with a 45–65% relative humidity and rotated 90° every 2 h. Four-day-incubated eggs were kept in the plasma reactor (Figure 1), exposing at different intensity and time following our previously described method [3,4]. Briefly, the non-thermal DBD plasma reactor had two disk-shaped electrodes (140 mm) and a glass dielectric barrier (5 mm) (Figure 1). Nineteen needles with a 2 mm thickness and 5 mm length were distributed on the surface of upper electrode. The gap between the needle tip and the eggshell near to the blastoderm was approximately 10 mm. Operating frequency of high voltage alternating current was 60 Hz. Argon flow rate was 2 L/min. The voltage was detected using a high voltage probe (1000×, P6015, Tektronix, Beaverton, OR, USA) and recorded by a digital oscilloscope (TBS1064, Tektronix). Plasma-treated fertilized eggs were incubated for 21 days and hatched out with a hatchability of approximately 80%. Chickens were housed in individual cages under the same environment conditions and given free access to equal amount of water and basic feed. Commercial crumbles (containing 18.00% crude protein, 2.50% crude fat, 7.00% crude fiber, 0.85% lysine, 0.25% methionine, 1.00% calcium, 0.70% phosphorus, and 0.50% salt) were used for feeding chicks aged at day 1 to 8 weeks. Commercial pellet feeds (containing 17.00% crude protein, 2.50% crude fat, 8.00% crude fiber, 0.40% lysine, 0.25% methionine, 1.00% calcium, 0.40% phosphorus, and 0.50% salt) were used for feeding adolescent chickens aged at 8 weeks to 13 weeks. There were no differences in feed intake for each group. Twenty fasted females and 20 males in each group were randomly used for measuring body weight on days 0 (the first day after hatching out), 30, 60, and 90. The 90-day-old chickens were photographed using a digital camera. The daily gain was calculated by dividing the body weight gain (final body weight − initial body weight) by the time period. The tibia length of chickens was measured. There was no mortality during the period of this study.

### 4.2. Serum Hormones, ATP, ROS, and Antioxidant Enzyme Analyses

The sera of chickens (10 females and 10 males in each group) on days 30, 60, and 90 were collected through wing vein and centrifuged for 20 min at 1000× *g*. Serum T3, T4, FT3, FT4, GH, IGF1, and IGFBP2 levels were detected following our previously described method [3]. Tissues obtained from the heart, liver, gizzard, intestine, and skeletal muscle of 90-day-old chickens after euthanasia were homogenized in 0.1% sodium dodecyl sulfate dissolved in PBS (0.05 mol/L, pH 7.4). The supernatants were collected after centrifuging for 10 min at 12,000× *g*. The protein concentration was measured using the bicinchoninic acid protein assay kit (Sigma-Aldrich, St. Louis, MO, USA). The serum and tissue homogenate supernatant were analyzed for ATP, SOD, CAT, GPx, ROS, and MDA concentrations following our previously described method [2,3,4].

### 4.3. Transmission Electron Microscopy and Mitochondrial Respiratory Enzyme Analyses

Small blocks of skeletal muscles of 90-day-old male chickens were placed in a mixture of 2.5% glutaraldehyde in PBS (0.05 mol/L, pH 7.4) for 24 h at 4 °C. Muscle blocks were subsequently post-fixed in 1% osmium tetroxide for 1 h, dehydrated in acetone, and embedded in Epon Araldite. Ultrathin sections (50–70 nm) were cut and mounted on copper grids, stained with uranyl acetate and lead citrate, and then photographed using the MegaView Soft Imaging System (Olympus, Tokyo, Japan) at 120 kV. The mitochondria of skeletal muscles obtained from 90-day-old male chickens were isolated and purified using Qproteome Mitochondria Isolation Kit (QIAGEN, Valencia, CA, USA) according to the manufacturer’s protocol. The mitochondrial respiratory enzyme concentrations were detected as our previously described method [2].

### 4.4. RT-PCR Analysis

Total RNA was isolated and purified from skeletal muscles and thyroid glands of 90-day-old chickens using TRIzol Reagent (Invitrogen, Thermo Fisher Scientific, Waltham, MA, USA). cDNA synthesis was performed using TOPscript^TM^ RT DryMIX (dT18) (Enzynomics, Daejeon, Korea). RT-PCR analysis was performed using Prime Taq Premix (2×) (GENETBIO, Yuseong-gu, Daejeon, Korea), and EvaGreen Dye (Biotium, Hayward, CA, USA) according to the manufacturer’s instructions. Primer sequences for RT-PCR are shown in Table 3. mRNA relative expression levels were normalized to the housekeeping gene (*β-actin*) and calculated using the 2^−ΔΔ*C*t^ method.

### 4.5. Methylation Sequencing

Genomic DNA was isolated and purified from skeletal muscles and thyroid glands of 90-day-old chickens using an AllPrep DNA/RNA Micro Kit (QIAGEN). Sodium bisulfite conversion bisulfite-sequencing PCR (BSP) was performed using the EpiTech Bisulfite Kit (QIAGEN). BSP primers were designed using MethPrimer (http://www.urogene.org/methprimer/) (Table 4). BSP products were purified, ligated, and transformed using the pGEM-T Easy Vector system I (Promega, Madison, WI, USA). Plasmids containing the target DNA were confirmed and sequenced as previously described method [2,5]. The cytosine methylation was analyzed using CyMATE software. The total methylation ratio and average methylation levels for CG, CHG and CHH were calculated as previously described method [2,5].

### 4.6. Western Blotting

Skeletal muscles of 90-day-old chickens were cut into small pieces and resuspended in RIPA lysis and extraction buffer (Thermo Fisher Scientific) for 30 min at 4 °C prior to centrifugation for 10 min at 12,000× *g*. The protein concentration was measured using a bicinchoninic acid protein assay kit (Sigma-Aldrich) and adjusted to equal protein concentration. Western blotting was performed as our previously described method [2,4,6,46]. The following antibodies were used: anti-phospho-AMPKα (Thr172, p-AMPKα; rabbit polyclonal; Cell Signaling Technology, Beverly, MA, USA; 1:1000), anti-AMPKα (rabbit polyclonal; Cell Signaling Technology; 1:1000), anti-phospho-mTOR (Ser2448, p-mTOR; rabbit monoclonal; Cell Signaling Technology; 1:1000), anti-mTOR (rabbit polyclonal; Cell Signaling Technology; 1:1000), anti-ATP5A (rabbit polyclonal; Abcam, Cambridge, UK; 1:250), anti-GHR (rabbit polyclonal; Bioss, Woburn, MA, USA; 1:500), anti-IGFBP2 (rabbit polyclonal; Bioss; 1:500), anti-PRX III (mouse monoclonal; Santa Cruz Biotechnology, Dallas, TX, USA; 1:200), anti-beta actin (rabbit polyclonal; Bioss; 1:1000), goat anti-mouse (Santa Cruz Biotechnology; 1: 5000), and goat anti-rabbit (Abcam; 1: 5000) IgG coupled to horseradish peroxidase conjugate. Band intensity was quantified using the ImageJ software (National Institutes of Health, Bethesda, MA, USA). The densitometric value of each p-AMPKα, AMPKα, p-mTOR, and mTOR band was normalized to the β-actin before calculating the p-AMPKα/AMPKα and p-mTOR/mTOR ratios. The densitometric values of the ATP5A, GHR, IGFBP2, and PRDX3 bands were also normalized to the relevant β-actin.

### 4.7. Statistical Analysis

Data are represented as mean ± standard deviation (SD) of three independent measurements. Statistical analyses were performed using the Statistical Package for the Social Sciences (SPSS version 16.0). Statistically significant differences were determined by the one-way ANOVA with a Fisher’s least significant difference (LSD) test. The values were considered significantly different at *p <* 0.05.

## 5. Conclusions

Four-day-incubated fertilized eggs treated with non-thermal plasma at 11.7 kV for 2 min have the highest growth rate in chickens after hatching. Especially, plasma-treated male chickens conspicuously grow faster than females. The growth-promoting effect of plasma in chickens was regulated by ROS homeostasis and the significant improvement of growth metabolism by increasing GH-IGF1 and their receptors expression, and thyroid hormones and ATP levels, which resulted from the regulation of demethylation levels of growth and hormone biosynthesis-related genes in the skeletal muscles and thyroid glands. Our findings provide an innovative approach that non-thermal DBD plasma treatment of fertilized eggs before hatching as a potentially viable and safe strategy for improving the growth rate in chickens. We have a bold hypothesis that the treatment of optimal plasma on embryonic stage may provide a potential practical application in raising animal productivity.

## Figures and Tables

**Table 1:** Effect of plasma exposure at different potential and duration on the average daily gain (ADG) and tibia length of chickens.

Gender	Group	ADG (Day 0–30) (g/d)	Average Tibia Length on Day 30 (cm)	ADG (Day 0–60) (g/d)	Average Tibia Length on Day 60 (cm)	ADG (Day 0–90) (g/d)	Average Tibia Length on Day 90 (cm)
Female	Control	7.66 ± 0.13 ^cd^	5.37 ± 0.05 ^d^	7.68 ± 0.06 ^d^	8.27 ± 0.05 ^fg^	8.81 ± 0.04 ^ef^	11.58 ± 0.13 ^de^
	11.7 kV–30 s	7.91 ± 0.10 ^bc^	5.60 ± 0.08 ^c^	7.95 ± 0.14 ^cd^	8.43 ± 0.12 ^ef^	8.93 ± 0.14 ^def^	11.81 ± 0.17 ^cd^
	11.7 kV–1 min	7.94 ± 0.07 ^bc^	5.70 ± 0.08 ^c^	10.04 ± 0.15 ^a^	9.13 ± 0.09 ^b^	9.66 ± 0.10 ^b^	11.99 ± 0.03 ^bc^
	11.7 kV–2 min	8.42 ± 0.28 ^b^	6.13 ± 0.05 ^b^	10.28 ± 0.31 ^a^	9.60 ± 0.08 ^a^	10.10 ± 0.09 ^a^	12.88 ± 0.04 ^a^
	16.4 kV–30 s	7.98 ± 0.08 ^bc^	5.67 ± 0.12 ^c^	8.03 ± 0.13 ^cd^	8.63 ± 0.09 ^de^	9.04 ± 0.11 ^cde^	11.89 ± 0.10 ^c^
	16.4 kV–1 min	8.07 ± 0.38 ^bc^	6.17 ± 0.05 ^b^	8.23 ± 0.18 ^c^	8.90 ± 0.08 ^c^	9.13 ± 0.20 ^cd^	12.01 ± 0.08 ^bc^
	16.4 kV–2 min	7.65 ± 0.17 ^cd^	5.60 ± 0.08 ^c^	8.02 ± 0.28 ^cd^	8.57 ± 0.05 ^de^	9.05 ± 0.08 ^cde^	11.76 ± 0.20 ^cd^
	22.0 kV–30 s	8.58 ± 0.60 ^b^	6.33 ± 0.17 ^b^	8.28 ± 0.02 ^c^	8.73 ± 0.05 ^cd^	9.13 ± 0.14 ^cd^	11.89 ± 0.05 ^c^
	22.0 kV–1 min	9.67 ± 0.49 ^a^	6.67 ± 0.12 ^a^	8.91 ± 0.22 ^b^	8.93 ± 0.12 ^bc^	9.24 ± 0.05 ^c^	12.20 ± 0.04 ^b^
	22.0 kV–2 min	7.60 ± 0.13 ^cd^	5.63 ± 0.05 ^c^	8.11 ± 0.30 ^cd^	8.20 ± 0.14 ^g^	8.75 ± 0.14 ^f^	11.48 ± 0.20 ^e^
	27.6 kV–30 s	8.22 ± 0.37 ^bc^	5.70 ± 0.08 ^c^	8.16 ± 0.10 ^c^	8.10 ± 0.08 ^gh^	8.43 ± 0.06 ^g^	11.34 ± 0.03 ^ef^
	27.6 kV–1 min	7.03 ± 0.18 ^de^	5.10 ± 0.16 ^e^	8.09 ± 0.23 ^cd^	7.97 ± 0.17 ^hi^	8.36 ± 0.09 ^g^	11.17 ± 0.06 ^fg^
	27.6 kV–2 min	6.79 ± 0.22 ^e^	5.03 ± 0.09 ^e^	7.92 ± 0.23 ^cd^	7.77 ± 0.05 ^i^	8.25 ± 0.20 ^g^	11.08 ± 0.11 ^g^
Male	Control	7.74 ± 0.16 ^d^	5.90 ± 0.08 ^d^	8.91 ± 0.12 ^f^	9.23 ± 0.12 ^e^	9.51 ± 0.19 ^ef^	12.93 ± 0.08 ^f^
	11.7 kV–30 s	8.72 ± 0.37 ^c^	6.27 ± 0.12 ^c^	10.06 ± 0.12 ^cde^	9.37 ± 0.12 ^de^	10.37 ± 0.05 ^bc^	13.11 ± 0.03 ^ef^
	11.7 kV–1 min	8.80 ± 0.35 ^c^	6.40 ± 0.08 ^c^	10.24 ± 0.16 ^cd^	9.57 ± 0.12 ^cd^	10.47 ± 0.13 ^b^	13.41 ± 0.11 ^cd^
	11.7 kV–2 min	10.39 ± 0.30 ^b^	6.83 ± 0.05 ^b^	12.17 ± 0.09 ^a^	10.57 ± 0.17 ^a^	11.65 ± 0.09 ^a^	14.52 ± 0.03 ^a^
	16.4 kV–30 s	8.98 ± 0.29 ^c^	6.40 ± 0.08 ^c^	10.07 ± 0.15 ^cde^	9.57 ± 0.12 ^cd^	9.89 ± 0.05 ^de^	13.39 ± 0.17 ^cd^
	16.4 kV–1 min	8.97 ± 0.48 ^c^	6.80 ± 0.08 ^b^	10.37 ± 0.23 ^c^	9.57 ± 0.09 ^cd^	9.93 ± 0.40 ^d^	13.50 ± 0.15 ^c^
	16.4 kV–2 min	8.33 ± 0.18 ^c^	6.20 ± 0.14 ^c^	10.18 ± 0.32 ^cd^	9.30 ± 0.08 ^e^	9.43 ± 0.14 ^f^	13.19 ± 0.13 ^de^
	22.0 kV–30 s	9.93 ± 0.21 ^b^	6.73 ± 0.17 ^b^	10.47 ± 0.27 ^bc^	9.43 ± 0.05 ^de^	9.51 ± 0.24 ^ef^	13.44 ± 0.10 ^cd^
	22.0 kV–1 min	11.32 ± 0.14 ^a^	7.03 ± 0.09 ^a^	10.83 ± 0.05 ^b^	9.70 ± 0.22 ^b^	10.06 ± 0.08 ^cd^	13.85 ± 0.07 ^b^
	22.0 kV–2 min	7.19 ± 0.18 ^def^	5.87 ± 0.05 ^d^	9.68 ± 0.13 ^e^	8.63 ± 0.05 ^f^	8.74 ± 0.13 ^g^	12.04 ± 0.20 ^g^
	27.6 kV–30 s	7.46 ± 0.31 ^de^	5.30 ± 0.08 ^e^	9.82 ± 0.22 ^de^	7.90 ± 0.08 ^g^	8.23 ± 0.09 ^h^	11.06 ± 0.11 ^h^
	27.6 kV–1 min	6.85 ± 0.29 ^ef^	5.13 ± 0.05 ^e^	8.82 ± 0.30 ^f^	7.83 ± 0.05 ^g^	8.10 ± 0.26 ^h^	10.97 ± 0.07 ^h^
	27.6 kV–2 min	6.68 ± 0.27 ^f^	5.10 ± 0.08 ^e^	6.04 ± 0.19 ^g^	7.73 ± 0.05 ^g^	7.25 ± 0.16 ^i^	10.22 ± 0.11 ^i^

Data are presented as mean ± standard deviation (SD) (*n* = 20) of three replicates; *n* represents an individual chicken. Within a column: different lowercase letters indicate significant differences (*p <* 0.05) in females and males, respectively, according to the one-way ANOVA with a least significant difference (LSD) test.

**Table 2:** DNA methylation levels (%).

Tissues	Genes	C-F	C-M	P-F	P-M
Skeletal muscles	*ATP5A1*	29.81	22.50	23.85	6.35
	*GH*	62.24	46.94	52.24	32.04
	*GHR*	54.33	44.17	47.33	30.67
	*IGF1*	61.79	53.57	54.64	42.50
	*IGF1R*	42.35	40.00	39.12	25.88
	*AMPKα2*	40.00	52.41	57.93	73.10
	*mTOR*	35.41	27.38	29.84	12.13
Thyroid glands	*TG*	47.42	41.82	30.61	20.61
	*THRA*	33.68	26.05	28.03	16.45

Genes in the skeletal muscles and thyroid glands of female and male chickens on day 90 are bisulfite sequenced. The cytosine methylation analysis results see Figure 8. C-F represents female chickens in the control group; C-M represents male chickens in the control group; P-F represents female chickens in the plasma treatment group; P-M represents male chickens in the plasma treatment group. *ATP5A1*, ATP synthase, H^+^ transporting, mitochondrial F1 complex, alpha subunit 1; *GH*, growth hormone; *GHR*, growth hormone receptor; *IGF1*, insulin-like growth factor 1; *IGF1R*, insulin-like growth factor 1 receptor; *AMPKα2*, adenosine monophosphate-activated protein kinase catalytic subunit alpha 2; *mTOR*, mammalian target of rapamycin; *TG*, thyroglobulin, *THRA*, thyroid hormone receptor alpha.

**Table 3:** Primer sequences for RT-PCR.

Gene	Sequence Number	Sequence Position	Product Length (bp)	Annealing Temperature (°C)	Sequence (5′to3′)
*β-actin*	NM_205518.1	625–818	194	57	F: GTGCGTGACATCAAGGAGAAGC
					R: CCACAGGACTCCATACCCAAGA
*ATP5A1*	NM_204286.1	1207–1364	158	57	F: GGTATCCGTCCAGCCATCAA
					R: GCATCCAAATCAGACCCAAACT
*ATP5B*	NM_001031391.2	482–637	156	57	F: GCCCCATCACAACGAAACAG
					R: CGCCTCCAAACAAACCAATC
*ATP5C1*	NM_001278096.1	272–411	140	57	F: ATTAAGGCACCCGAGGACAA
					R: ACTTCCTTCCCTGCATTGGA
*ATP5F1*	XM_417993.4	437–644	208	57	F: CATTGGAGACTGCCATTGAGG
					R: TGATCTTGCTCTTTCTGACGCTT
*ATP5G1*	XM_001233602.3	287–536	250	57	F: CAGGAGCAGGTATTGGGACA
					R: TTGTCAGTCTGGAACGCTCT
*ATP5G3*	NM_001277855.1	141–288	148	57	F: CCAAAACGCTGTCTCCCAAC
					R: ACCGAAGACCGTTCCAATACC
*ATP5H*	XM_001232598.3	332–551	220	57	F: CTGAAGGTCCCTGAACCAGT
					R: ACTTCTCCCTGTCCAGTCTG
*ATP5I*	NM_001097534.2	74–240	167	57	F: TCTCGCCCCTCATCAAGTTC
					R: TGCCAGTTCCTTTGCAATCC
*ATP5J*	XM_004938370.1	58–197	140	58	F: CACTTGCGGAGAAACATCGGT
					R: CCTACATCAACAGGTCCTCCAGC
*ATP5J2*	NM_001257200.1	170–263	94	57	F: GCCTCGGTGGTATCAGTATGGT
					R: TACTTCCTGCGGCGGTCAT
*ATP5L*	XM_015298211	250–377	128	57	F: CCATGGTCAGGAGCTTTCAG
					R: GCCTCGTTTGCCTATGATCTC
*ATP5S*	NM_001277562.1	46–279	234	57	F: TCCCCTTCCCCTTTCTTTCC
					R: CATAGCCTTGATAGCGCACC
*GH*	NM_204359.2	104–284	181	57	F: TGTTTGCCAACGCTGTGCT
					R: TTCTGCTGGGCGTCATCCT
*GHR*	NM_001001293.1	1070–1299	230	57	F: GTCACACAGTTGCTTGGGAG
					R: TATGCGGCTGTTGGGTATCT
*IGF1*	NM_001004384.2	188–316	129	58	F: AGTTCGTATGTGGAGACAGAGGC
					R: CCAGCCTCCTCAGGTCACAAC
*IGF1R*	NM_205032.1	2961–3114	154	57	F: TTGTGCTCCCCATTGCTTTC
					R: GGAACGTACACATCCGAAGC
*IGFBP2*	NM_205359.1	582–793	212	57	F: TCACAACCACGAGGACTCAAAG
					R: GCTGCCCATTCACCGACAT
*POU1F1*	NM_204319.1	560–754	195	57	F: ATGTTGGCGAAGCACTGGC
					R: GCTTCCTCTTCCGCTCATTCA
*AMPKα2*	NM_001039605.1	726–943	218	57	F: GGAGGCGTGTTTTACATCCC
					R: AACTTCTCACAGACCTCCCG
*AMPKβ2*	NM_001044662.1	435–661	227	57	F: CCAGTGTTTTCAGCTCCCAC
					R: GAGGTCCAGGATAGCGACAA
*AMPKγ3*	NM_001031258.2	183–320	138	57	F: GCTGGAACCCGACAACAATT
					R: GCCTTCTTGATCTCCAGGGT
*mTOR*	XM_417614.4	119–309	191	57	F: TGAAGGGGTCAAGGCAATCC
					R: GGCGAGCAGTGGTTGTGGAT
*PRDX1*	NM_001271932.1	358–545	188	56	F: ACAAGGTGGTTTGGGCACTA
					R: TCTCATCAACAGAACGGCCA
*PRDX3*	XM_426543.5	414–551	138	56	F: TTTCACCTTTGTGTGCCCCA
					R: TTGCGCGGGGTATTTATCCA
*PRDX4*	XM_001233999.3	595–733	139	56	F: TGCACTTAGGGGCCTTTTCA
					R: TTCTCCATGCTTGTCCGTGT
*PRDX6*	NM_001039329.2	189–340	152	58	F: TGAGTTCAGCAAACGCAACG
					R: GCTCTCGGTCCTTATCAGCG
*TG*	XM_015283114.1	2812–2966	155	57	F: GCAGCTTTCCAAACCTTCAG
					R: GGCTCCAGCACAGAGAAAAC
*TPO*	XM_015284944.1	1872–2075	204	58	F: TGACGCTCAAAAGCATGAAC
					R: TGCTTTGGTGTTCCACACAT
*THRA*	NM_205313.1	355–636	282	57	F: AAGCGCAAAAGAAAGAGCAG
					R: CACGGAGATGCACTTCTTGA
*THRB*	NM_205447.2	976–1150	175	58	F: TTTCCTCCTGGCATTTGAAC
					R: CAGGAACAATGGAGGGAAGA

*ATP5B*, ATP synthase, H^+^ transporting, mitochondrial F1 complex, beta polypeptide; *ATP5C1*, ATP synthase, H^+^ transporting, mitochondrial F1 complex, gamma polypeptide 1; *ATP5F1*, ATP synthase, H^+^ transporting, mitochondrial Fo complex, subunit B1; *ATP5G1*, ATP synthase, H^+^ transporting, mitochondrial Fo complex, subunit C1; *ATP5G3*, ATP synthase, H^+^ transporting, mitochondrial Fo complex, subunit C3; *ATP5H*, ATP synthase, H^+^ transporting, mitochondrial Fo complex, subunit D; *ATP5I*, ATP synthase, H^+^ transporting, mitochondrial Fo complex, subunit E; *ATP5J*, ATP synthase, H^+^ transporting, mitochondrial Fo complex, subunit F6; *ATP5J2*, ATP synthase, H^+^ transporting, mitochondrial Fo complex, subunit F2; *ATP5L*, ATP synthase, H^+^ transporting, mitochondrial Fo complex, subunit G; *ATP5S*, ATP synthase, H^+^ transporting, mitochondrial Fo complex, subunit S; *IGFBP2*, insulin-like growth factor binding protein 2; *POU1F1*, POU class 1 homeobox 1; *AMPKβ2*, adenosine monophosphate-activated protein kinase non-catalytic subunit beta 2; *AMPKγ3*, adenosine monophosphate-activated protein kinase non-catalytic subunit gamma 3; *PRDX*, peroxiredoxin; *TPO*, thyroid peroxidase; *THRB*, thyroid hormone receptor beta.

**Table 4:** Primer sequences for bisulfite-sequencing PCR.

Gene	Chromosome Location	Sequence Position	Product Length (bp)	Annealing Temperature (°C)	Sequence (5′to3′)	Expected No. of CpGs
*ATP 5A1*	Z	1082–1292	211	55	F: GAGGTTTTTTGATTGTTTTGTTTGT	4
					R: CCTACCACCTATTTCATTACCCTAAT	
*GH*	27	458–611	154	53	F: AAGAAGGGATTTAAGTTTTGATGAG	10
					R: TCCTTCTTAAAACAAAACAACAAAC	
*GHR*	Z	436–768	333	53	F: GTTAAATTGGGATTTATGGGGATAT	6
					R: CAACAACTAAAAACCAAAAAAACTC	
*IGF1*	1	413–661	249	53	F: TTGAGTTGGTTGATGTTTTTTAGTT	5
					R: TCAAATACACTTCCTTTTATACTTTTAACA	
*IGF1R*	10	308–554	247	52	F: GTTGAGGGTTTTTTTTAGATTTTGTT	12
					R: TTATATTCCTCAAATTATAAAACCC	
*AMPKα2*	8	233–463	231	52	F: AGAAATTTAAAATTTGAAATTTTTT	10
					R: AAATCCCTATAAACAACCATATATC	
*mTOR*	21	1690–1890	201	52	F: ATATTTAGGATGGGTTGTTGAAAAT	4
					R: AAATCAAAAAATACCCTTCAAACTC	
*TG*	2	2943–3144	202	53	F: TATTGTTTTTTTTGTGTTGGAGTTT	12
					R: TATATATCCCCCATCAATATCACAC	
*THRA*	27	145–385	241	51	F: GGGATGTTGTAGGGAGTTTAGTATT	19
					R: CCAAACATTAACTACTCTTTCTTTTAC	

## Supplementary Materials

Supplementary Materials can be found at http://www.mdpi.com/1422-0067/19/8/2301/s1.

## Abbreviations

DBD	Dielectric barrier discharge
ROS	Reactive oxygen species
ADG	Average daily gain
T3	Triiodothyronine
T4	Thyroxine
FT3	Free triiodothyronine
FT4	Free thyroxine
GH	Growth hormone
IGF1	Insulin-like growth factor 1
IGFBP2	Insulin-like growth factor binding protein 2
ATP	Adenosine triphosphate
SOD	Superoxide dismutase
CAT	Catalase
GPx	Glutathione peroxidase
MDA	Malondialdehyde
NADH	Nicotinamide adenine dinucleotide hydrogen
GHR	Growth hormone receptor
IGF1R	Insulin-like growth factor 1 receptor
POU1F1	POU class 1 homeobox 1
mTOR	Mammalian target of rapamycin
AMPK	Adenosine monophosphate-activated protein kinase
PRDXs	Peroxiredoxins
TG	Thyroglobulin
TPO	Thyroid peroxidase
THRs	Thyroid hormone receptors
BSP	Bisulfite-sequencing PCR
SD	Standard deviation
LSD	Fisher’s least significant difference
