# Supplementary material for: Innovative Approach of Non-Thermal Plasma Application for Improving the Growth Rate in Chickens
Source: Int J Mol Sci. 2018 Aug 6;19(8):2301. doi: 10.3390/ijms19082301 (PMC6121326; doi:10.3390/ijms19082301)
Supplement: Supplementary file 1 [file ijms-19-02301-s001.pdf]

Supplementary Materials

Supplementary Figures

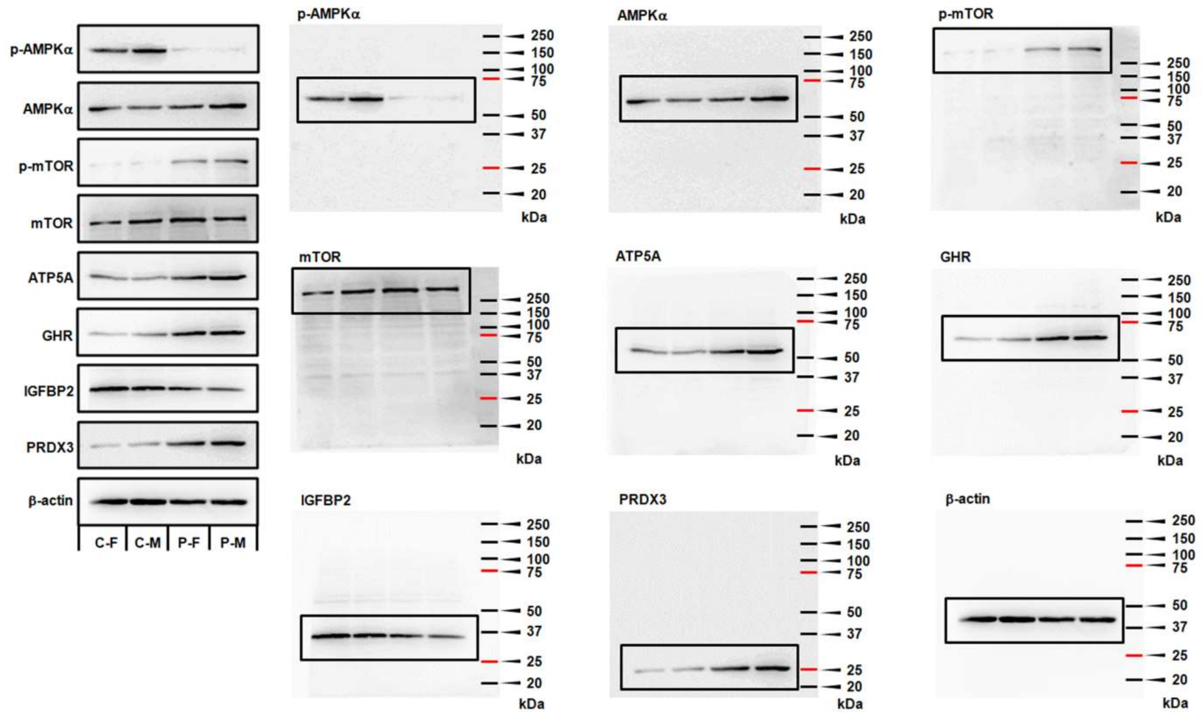

Figure S1. Scans of all immunoblots for Figure 7a. The grouping of gels/blots cropped from different gels. All blots are visualized with 5 min exposure time.
